# High maternal early-pregnancy blood glucose levels are associated with altered fetal growth and increased risk of adverse birth outcomes

**DOI:** 10.1007/s00125-019-4957-3

**Published:** 2019-08-08

**Authors:** Madelon L. Geurtsen, Eef E. L. van Soest, Ellis Voerman, Eric A. P. Steegers, Vincent W. V. Jaddoe, Romy Gaillard

**Affiliations:** 1 The Generation R Study Group (Na 29-15), Erasmus MC, University Medical Center Rotterdam, PO Box 2040, 3000 CA Rotterdam, the Netherlands; 2 Department of Pediatrics, Sophia Children’s Hospital, Erasmus MC, University Medical Center Rotterdam, Rotterdam, the Netherlands; 3 Department of Health Sciences, Prevention and Public Health, VU Amsterdam, Amsterdam, the Netherlands; 4 Department of Obstetrics, Erasmus MC, University Medical Center Rotterdam, Rotterdam, the Netherlands; 5 Department of Epidemiology, Erasmus MC, University Medical Center Rotterdam, Rotterdam, the Netherlands

**Keywords:** Adverse birth outcomes, Fetal growth, Gestational diabetes mellitus, Maternal glucose, Maternal hyperglycaemia, Prospective cohort

## Abstract

**Aims/hypothesis:**

The study aimed to assess the associations of maternal early-pregnancy blood glucose levels with fetal growth throughout pregnancy and the risks of adverse birth outcomes.

**Methods:**

In a population-based prospective cohort study among 6116 pregnant women, maternal non-fasting glucose levels were measured in blood plasma at a median 13.2 weeks of gestation (95% range 9.6–17.6). We measured fetal growth by ultrasound in each pregnancy period. We obtained information about birth outcomes from medical records and maternal sociodemographic and lifestyle factors from questionnaires.

**Results:**

Higher maternal early-pregnancy non-fasting glucose levels were associated with altered fetal growth patterns, characterised by decreased fetal growth rates in mid-pregnancy and increased fetal growth rates from late pregnancy onwards, resulting in an increased length and weight at birth (*p* ≤0.05 for all). A weaker association of maternal early-pregnancy non-fasting glucose levels with fetal head circumference growth rates was present. Higher maternal early-pregnancy non-fasting glucose levels were also associated with an increased risk of delivering a large-for-gestational-age infant, but decreased risk of delivering a small-for-gestational-age infant (OR 1.28 [95% CI 1.16, 1.41], OR 0.88 [95% CI 0.79, 0.98] per mmol/l increase in maternal early-pregnancy non-fasting glucose levels, respectively). These associations were not explained by maternal sociodemographic factors, lifestyle factors or BMI. Maternal early-pregnancy non-fasting glucose levels were not associated with preterm birth or delivery complications.

**Conclusions/interpretation:**

Higher maternal early-pregnancy non-fasting glucose levels are associated with decreased fetal growth rates in mid-pregnancy and increased fetal growth rates from late pregnancy onwards, and an increased risk of delivering a large-for-gestational-age infant. Future preventive strategies need to focus on screening for an impaired maternal glucose metabolism from preconception and early pregnancy onwards to improve birth outcomes.

**Electronic supplementary material:**

The online version of this article (10.1007/s00125-019-4957-3) contains peer-reviewed but united supplementary material, which is available to authorised users.

## Introduction

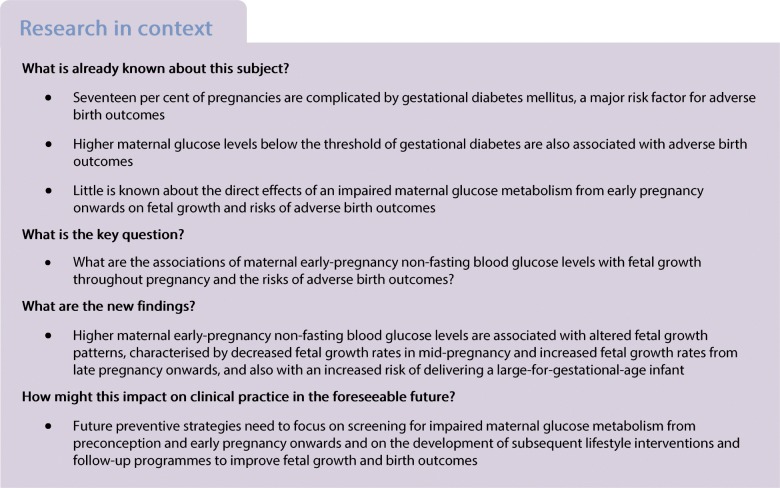



Gestational diabetes mellitus (GDM) complicates up to 17% of pregnancies and is a major risk factor for maternal and fetal perinatal complications [1–3]. Recent studies suggest that these associations are also present for higher maternal glucose levels below the threshold of GDM [4–6]. A meta-analysis of 25 prospective studies showed that higher maternal glucose levels in mid-pregnancy and late pregnancy are related to increased risks of perinatal complications [7].

Accumulating evidence suggests that early pregnancy is a critical period for the effects of adverse exposures on embryonic and placental development [8, 9]. Little is known, however, about the direct effects of an impaired maternal glucose metabolism from early pregnancy onwards on fetal growth and the risks of adverse birth outcomes in both diabetic and non-diabetic pregnant women [3]. Among women with GDM, fetal growth may already be abnormal preceding this diagnosis. However, results are inconsistent and difficult to interpret as maternal glucose levels before the diagnosis of GDM are unknown [10, 11]. We hypothesised that a maternal glucose metabolism already impaired in early pregnancy affects embryonic and placental development, subsequently leading to altered fetal growth and increased risks of adverse birth outcomes [2, 3, 12, 13]. Insight into the influence of maternal blood glucose levels from early pregnancy onwards on fetal development is important, as maternal blood glucose levels offer a major target for potential future interventions.

Therefore, in a population-based prospective cohort study among 6116 pregnant women, we examined whether maternal early-pregnancy non-fasting glucose levels across the full range, and not limited to diagnostic thresholds, are associated with fetal growth in each pregnancy period and with the risks of adverse birth outcomes. To obtain further insight into the causality of these associations, we additionally explored whether these associations are explained by maternal sociodemographic factors or lifestyle factors.

## Methods

### Study design

**Fig. 1:**
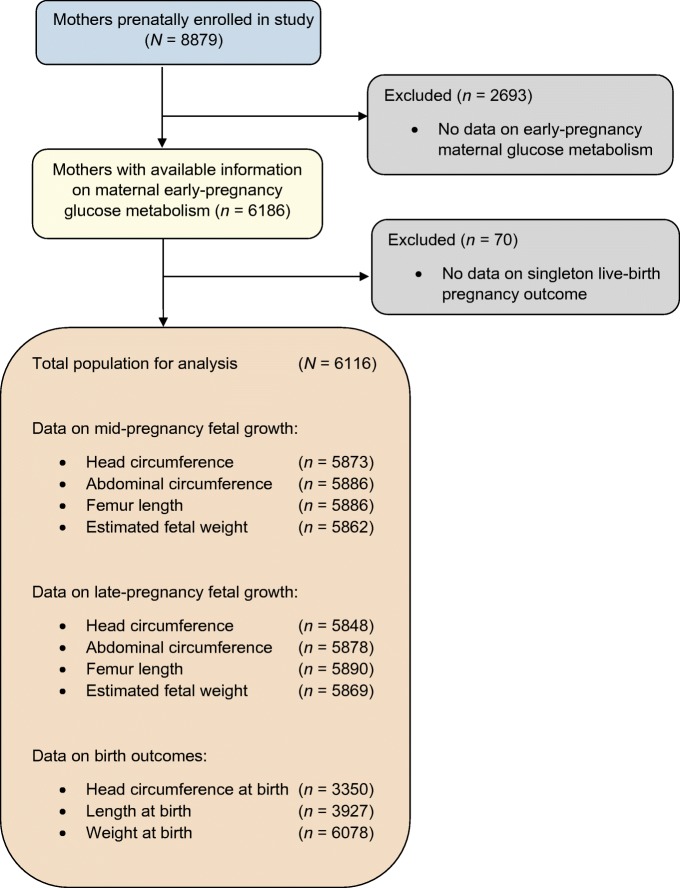
Study participant flow chart

This study was embedded in the Generation R Study, a population-based prospective cohort study from early pregnancy onwards in Rotterdam, the Netherlands [14]. The study was approved by the local Medical Ethical Committee (MEC 198.782/2001/31). Written informed consent was obtained from all participating women. All pregnant women, who were resident in the study area at their delivery date, were enrolled between 2001 and 2005. Translated information packages and questionnaires were available for recruitment of different ethnicities. The enrolment procedure has been described in detail previously [15]. Response rate at birth was 61% [16]. In total, 8879 women were enrolled during pregnancy, of whom 6186 had measurements of glucose levels available. We excluded pregnancies not leading to singleton live births (*n* = 70). The population for analyses comprised 6116 women (Fig. 1). Since only a small number of woman (*n* = 24) had pre-gestational diabetes mellitus, these individuals were included in the analyses. A sensitivity analysis excluding women with pre-gestational diabetes mellitus was performed.

### Maternal glucose metabolism

Blood samples were collected once in early pregnancy at 13.2 median weeks’ gestation (95% range 9.6–17.6). After 30 min of fasting, venous blood samples were collected from pregnant women by research nurses and temporally stored at room temperature. We considered the 30 min fasting samples non-fasting samples. This time-interval was chosen because of the design of our study, in which it was not possible to obtain fasting samples from all pregnant women. At least every 3 h, blood samples were transported to a dedicated laboratory facility of the regional laboratory in Rotterdam, the Netherlands (Star-MDC), for further processing and storage [17]. Glucose (mmol/l) is an enzymatic quantity and was measured with the c702 module on a Cobas 8000 analyser. Insulin (pmol/l) was measured with electrochemiluminescence immunoassay on a Cobas e411 analyser. Quality control samples demonstrated intra- and inter-assay CVs of 1.30% and 2.50%, respectively. We constructed maternal early-pregnancy non-fasting glucose and insulin SD scores (SDSs).

Information on pre-gestational diabetes mellitus was obtained from self-reported questionnaires and on GDM from medical records after delivery. GDM was diagnosed by a community midwife or an obstetrician according to Dutch midwifery and obstetric guidelines using the following criteria: either a random glucose level >11.0 mmol/l, a fasting glucose ≥7.0 mmol/l or a fasting glucose between 6.1 and 6.9 mmol/l with a subsequent abnormal GTT [18]. In clinical practice and for this study sample, an abnormal GTT was defined as a glucose level greater than 7.8 mmol/l after glucose intake.

### Fetal growth patterns and adverse birth outcomes

Fetal ultrasound examinations were carried out in two dedicated research centres in early pregnancy (13.2 median weeks’ gestation [95% range 9.6–17.6]), mid-pregnancy (20.5 median weeks’ gestation [95% range 18.7–23.1]) and late pregnancy (30.3 median weeks’ gestation [95% range 28.5–32.8]). In early pregnancy we used crown–rump length to assess fetal growth only in mothers with a known and reliable first day of the last menstrual period, a regular menstrual cycle of 28 days (range 24–32 days) and who had fetal crown–rump length measured between a gestational age of 10 weeks 0 days and 13 weeks 6 days (*n* = 1470), as described previously [19]. The first day of the last menstrual period was obtained from the referring letter from the community midwife or hospital. This date was confirmed with the participants at the ultrasound visit and additional information on the regularity and duration of the menstrual cycle was obtained [19]. For mothers without this information, gestational age was established by early- pregnancy fetal ultrasound examination. This strategy was performed because of the large number of mothers who did not know the exact date of their last menstrual period or who had irregular menstrual cycles [20]. Subsequently, in mid-pregnancy and late pregnancy, we measured fetal head circumference, abdominal circumference and femur length to the nearest millimetre using standardised ultrasound procedures. Estimated fetal weight was subsequently calculated using the formula of Hadlock et al [21]. Longitudinal growth curves and gestational-age-adjusted SDSs were constructed for all fetal biometry measurements [20]. These gestational-age-adjusted SDSs were based on reference growth curves from the whole study population and represent the equivalent of *z* scores [20].

Information about offspring sex, gestational age, weight, length and head circumference at birth was obtained from medical records [14]. Since head circumference and length were not routinely measured at birth, fewer measurements were available (*n* = 3350 for head circumference and *n* = 3927 for length at birth). Gestational-age-adjusted SDSs for head circumference, length and weight at birth were constructed using North European growth standards as the reference growth curve and represent the equivalent of *z* scores [22]. Small-for-gestational-age and large-for-gestational-age at birth were defined as the lowest and highest 10 percentiles of gestational age- and sex-adjusted birthweight using North European growth standards [22]. Preterm birth was defined as a gestational age at birth <37 weeks. Information on delivery complications, Caesarean delivery and vacuum extraction, was collected from medical records.

### Covariates

Information on maternal age, pre-pregnancy weight, educational level, ethnicity, parity and folic acid supplements use was obtained at enrolment by questionnaires [14]. Height and weight, both without shoes and heavy clothing, were measured at enrolment. Pre-pregnancy BMI was calculated (self-reported pre-pregnancy weight in kilograms divided by height measured at first study visit in metres squared). Information about smoking and alcohol consumption was assessed by questionnaires. We dichotomised both variables; women were classified as ‘yes’ if they consumed alcohol and smoked until pregnancy was known and if they continued to drink alcohol and smoke throughout pregnancy. Information on total daily energy intake was obtained by a food frequency questionnaire in early pregnancy [14].

### Statistical analysis

First, we conducted a non-response analysis to compare characteristics of women with and without glucose measurements available. Normally distributed data were presented as mean with SD; non-normally distributed data were presented as median with 95% range (i.e. the 2.5th to 97.5th percentile).

Second, we assessed the associations of maternal early-pregnancy non-fasting glucose levels with repeatedly measured fetal biometry measurements to assess fetal growth patterns using unbalanced repeated measurement regression models. These models take the correlation between repeated measurements of the same individual into account and allow for incomplete outcome data [23]. We included maternal early-pregnancy non-fasting glucose levels in these models as intercept and as interaction term with gestational age to estimate fetal growth rates over time [23]. These analyses were conducted without adjustment for covariates, which most clearly reflects clinical practice [7].

Third, we examined the associations of maternal early-pregnancy non-fasting glucose levels with detailed fetal biometry measurements in gestational-age-adjusted SDS in each pregnancy period using linear regression models. Analyses were repeated using fetal biometry measurements in absolute values. For these analyses, we constructed different models to explore whether these observed associations were explained by maternal sociodemographic and lifestyle factors: a basic model (adjusted for gestational age at assessment), a maternal ethnicity model (basic model additionally adjusted for ethnicity), a maternal pregnancy-related factors model (maternal ethnicity model additionally adjusted for maternal age, parity, educational level, daily total energy intake, smoking, alcohol consumption and folic acid supplement use) and a maternal BMI model (maternal pregnancy-related factors model additionally adjusted for maternal pre-pregnancy BMI). Included covariates were based on previous studies, strong correlations with maternal glucose levels, risk of GDM and fetal biometry measurements, and changes in effect estimates of >10% [2, 3].

Fourth, we assessed the associations of maternal early-pregnancy non-fasting glucose levels with the risks of adverse birth outcomes using multiple logistic regression models using the same adjustment models. We explored whether associations were non-linear by performing quintiles analyses and adding a quadratic term to the original model. However, for all analyses, a linear model had the best fit. Since only seven women had glucose levels of >7.8 mmol/l and only 62 women developed GDM, we were unable to explore the effects of these clinical categories on fetal growth and adverse birth outcomes. We tested but did not observe statistical interactions between maternal ethnicity or pre-pregnancy BMI and maternal early-pregnancy non-fasting glucose levels for the associations with fetal biometry measurements and with adverse birth outcomes [2, 3, 10, 11].

As a sensitivity analysis, analyses were repeated using maternal early-pregnancy non-fasting insulin levels. To enable comparison of effect sizes for the associations of different measures of maternal early-pregnancy glucose metabolism with fetal growth and birth outcomes, these sensitivity analyses were performed using maternal early-pregnancy non-fasting glucose and insulin levels in SDSs. In addition, we explored whether our observed associations were affected by specific subgroups. We performed five additional sensitivity analyses for the associations of maternal early-pregnancy non-fasting glucose levels with fetal biometry measurements in each pregnancy period: (1) excluding women with pre-gestational diabetes mellitus (*n =* 24); (2) excluding women with GDM (*n* = 62); (3) among women of Dutch ethnicity only; (4) among women included in early pregnancy only (before 14 weeks’ gestation); and (5) among term births only.

Missing data of covariates were imputed using multiple imputation. Five imputed datasets were created and analysed together. Repeated measurement analyses were performed using the Statistical Analysis System version 9.4 (SAS Institute, Cary, NC, USA; Proc Mixed module). All other analyses were performed using the Statistical Package of Social Sciences version 24.0 for Windows (SPSS, Chicago, IL, USA).

## Results

Population characteristics are shown in Table 1. Fetal growth characteristics of the study population are shown in Table 2. Non-response analyses showed that women without glucose measurements had a higher BMI, had a lower level of educational attainment, were of non-European descent and used folic acid supplements less often (electronic supplementary material [ESM] Table 1).

**Table 1:** Maternal and birth characteristics of the study population

Characteristic	Total group, *N* = 6116
Maternal characteristics	
Age, years	29.8 ± 5.1
Height, cm	167.5 ± 7.4
Pre-pregnancy weight, kg	64.0 (48.0–99.7)
Pre-pregnancy BMI, kg/m^2^	22.6 (18.0–34.7)
Gestational age at intake, weeks	13.2 (9.6–17.6)
Parity (nulliparous)	3474 (57.3)
Ethnicity	
Dutch	3083 (52.2)
European	496 (8.4)
Cape Verdean	245 (4.2)
Moroccan	353 (6.0)
Dutch Antillean	171 (2.9)
Surinamese	506 (8.6)
Turkish	474 (8.1)
Other	545 (9.3)
Education, higher	2550 (44.9)
Total energy intake, kJ	486 (134)
Folic acid use	
No	1183 (25.3)
Start first 10 weeks	1491 (31.9)
Start periconceptional	1997 (42.8)
Smoking during pregnancy, continued	1012 (18.6)
Alcohol use during pregnancy, continued	2095 (39.0)
Gestational hypertensive disorders	
Pre-eclampsia	127 (2.2)
Gestational hypertension	234 (4.1)
Glucose, mmol/l	4.4 ± 0.8
Insulin, pmol/l	114.6 (17.6–716.1)
Impaired glucose tolerance at intake^a^	17 (0.3)
Pre-gestational diabetes mellitus	24 (0.5)
GDM	62 (1.1)
Birth characteristics	
Male	3100 (50.7)
Gestational age, weeks	40.1 (35.6–42.3)
Preterm birth^b^	310 (5.1)
Small for gestational age^c^	606 (10.0)
Large for gestational age^d^	606 (10.0)
Caesarean delivery	692 (14.5)
Vacuum extraction	774 (15.9)

Values are numbers (%), means ± SD or medians (95% range)

^a^Impaired glucose tolerance at intake is defined as >7.8 mmol/l in non-fasting state

^b^Preterm birth is defined as <37 weeks’ gestation

^c^Small for gestational age is defined as <10th percentile of age- and sex-adjusted birthweight

^d^Large for gestational age is defined as >90th percentile of age- and sex-adjusted birthweight

**Table 2:** Fetal growth characteristics of the study population

Fetal growth characteristic	Total group, *N* = 6116
Mid-pregnancy	
Gestational age, weeks	20.5 (18.7–23.1)
Head circumference, mm	179 ± 13.3
Abdominal circumference, mm	156 ± 13.7
Femur length, mm	33 ± 3.3
Estimated fetal weight, g	377 ± 83.9
Late pregnancy	
Gestational age, weeks	30.3 (28.5–32.8)
Head circumference, mm	285 ± 12.2
Abdominal circumference, mm	264 ± 16.3
Femur length, mm	57 ± 8.7
Estimated fetal weight, g	1611 ± 251.0
Birth	
Gestational age, weeks	40.1 (35.6–42.3)
Birth head circumference, cm	33.8 ± 1.7
Birth length, cm	50.2 ± 2.4
Birthweight, g	3418 ± 563

Values are means ± SD or medians (95% range)

### Maternal blood glucose levels and fetal growth

**Fig. 2:**
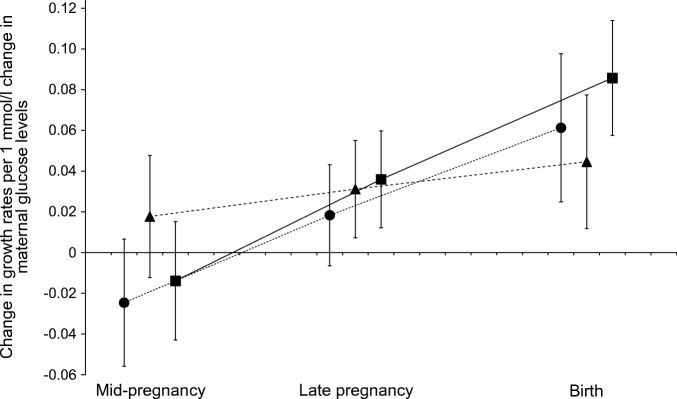
Differences in fetal growth rates per change in glucose levels. Data are SDS values (95% CI) from repeated measurement regression models that reflect the differences in gestational age-adjusted growth rates in SDS of head circumference (circles), length (triangles) and weight (squares) at mid-pregnancy, late pregnancy and at birth per 1 mmol/l change in maternal early-pregnancy glucose levels. As a measure of skeletal length growth from mid-pregnancy onwards, we used fetal femur length SDS in mid-pregnancy and late pregnancy and total body length SDS at birth within the repeated measurements model. All fetal biometry measurements for each pregnancy period were taken at the same time point. The models were adjusted for gestational age at intake. *p* <0.05 for interaction with gestational age for all models

Figure 2 shows that higher maternal early-pregnancy non-fasting glucose levels were associated with increased rates of fetal length and weight growth from late pregnancy onwards, resulting in increased length and weight at birth (*p* value for interaction with gestational age <0.05). Weaker effect estimates were observed for the associations of maternal early-pregnancy non-fasting glucose levels with rate of fetal head circumference growth, but a significant interaction with gestational age was also present (*p* value for interaction with gestational age <0.05).

**Fig. 3:**
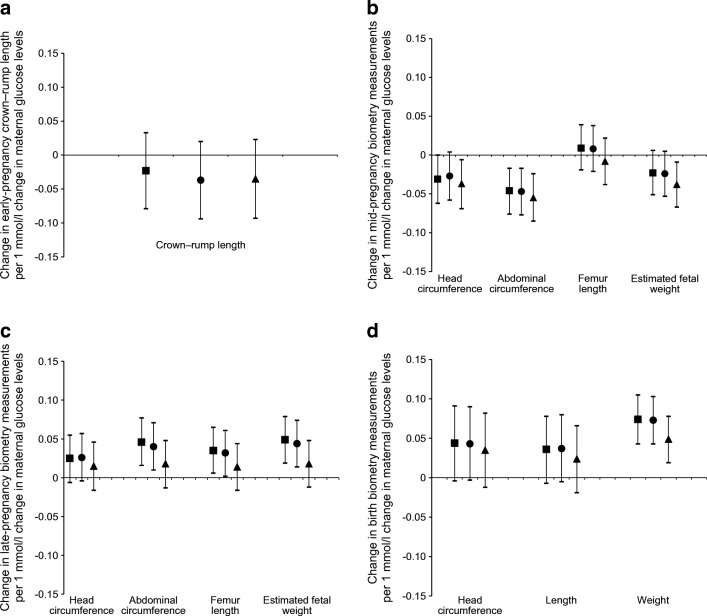
Associations of maternal early-pregnancy glucose levels in mmol/l with fetal biometry measurements (*N* = 6116). Data are SDS values (95% CI) from linear regression models that reflect the differences in growth characteristics in SDSs in (**a**) early pregnancy, (**b**) mid-pregnancy, (**c**) late pregnancy and (**d**) at birth, per 1 mmol/l change in maternal early-pregnancy glucose levels. Analyses with crown–rump length were based on subgroup analyses (*n* = 1470). Estimates are from multiple imputed data. Squares show basic model: adjusted for gestational age at assessment. Circles show maternal pregnancy-related factors model: basic model additionally adjusted for maternal ethnicity, age, parity, educational level, daily total energy intake, smoking, alcohol consumption and folic acid supplement use. Triangles show BMI model: maternal pregnancy-related factors model additionally adjusted for maternal pre-pregnancy BMI

Figure 3 shows that maternal early-pregnancy non-fasting glucose levels were not significantly associated with early-pregnancy fetal crown–rump length. Higher maternal early-pregnancy non-fasting glucose levels were associated with decreased mid-pregnancy fetal head circumference SDS and abdominal circumference SDS (*p* ≤0.05 for both). The association of higher maternal early-pregnancy non-fasting glucose levels with decreased mid-pregnancy estimated fetal weight SDS did not reach statistical significance (*p* = 0.10). No association with mid-pregnancy femur length was present. However, higher maternal early-pregnancy non-fasting glucose levels were associated with increased fetal head circumference SDS, abdominal circumference SDS, femur length SDS and estimated fetal weight SDS in late pregnancy, and head circumference SDS, length SDS and weight SDS at birth (*p* ≤0.05 for all). These associations were not explained by adjustment for maternal ethnicity, or other maternal pregnancy-related factors, but were partly attenuated after adjustment for maternal pre-pregnancy BMI (maternal ethnicity-adjusted model is given in ESM Table 2). The strongest effect estimate was present for birthweight (difference in birthweight in the maternal pregnancy-related model: 0.07 SDS [95% CI 0.04, 0.10] per mmol/l increase in maternal early-pregnancy non-fasting glucose levels; *p* ≤0.05). The associations of maternal early-pregnancy non-fasting glucose levels with absolute values of fetal biometry measurements are given in ESM Table 3 and showed similar findings to the main findings. Per 1 mmol/l increase in maternal early-pregnancy non-fasting glucose levels, birthweight increased by 25.4 g (95% CI 9.0, 41.8) in the maternal pregnancy-related model (*p* ≤0.05).

### Impact of maternal early-pregnancy blood glucose levels on adverse birth outcomes

Independent of maternal sociodemographic or lifestyle factors, higher maternal early-pregnancy non-fasting glucose levels were associated with an increased risk of delivering a large-for-gestational-age infant, but with a decreased risk of delivering a small-for-gestational-age infant (ORs 1.28 [95% CI 1.16, 1.41] and 0.88 [95% CI 0.79, 0.98] per mmol/l increase in maternal early-pregnancy non-fasting glucose levels in the maternal pregnancy-related model [*p* ≤0.05], respectively) (Table 3, and maternal ethnicity-adjusted model is given in ESM Table 4). No significant associations were present for maternal early-pregnancy non-fasting glucose levels with preterm birth, Caesarean delivery or vacuum extraction*.*

**Table 3:** Associations of maternal early-pregnancy glucose levels with the risks of adverse birth outcomes

Maternal early-pregnancy glucose levels (mmol/l)	Small for gestational age at birth	Large for gestational age at birth	Preterm birth	Caesarean delivery	Vacuum extraction
Basic model^a^	0.89 (0.80, 0.98)*	1.27 (1.16, 1.40)*	1.08 (0.95, 1.24)	1.12 (0.98, 1.27)	0.99 (0.90, 1.09)
Maternal pregnancy-related factors model^b^	0.88 (0.79, 0.98)*	1.28 (1.16, 1.41)*	1.08 (0.94, 1.23)	1.11 (1.00, 1.23)	1.01 (0.91, 1.12)
BMI model^c^	0.91 (0.82, 1.02)	1.21 (1.10, 1.34)*	1.06 (0.92, 1.21)	1.09 (0.99, 1.20)	1.01 (0.90, 1.12)

Values are ORs (95% CI) from logistic regression models that reflect the differences in risks of adverse birth outcomes per 1 mmol/l increase in maternal early-pregnancy glucose levels. Estimates are from multiple imputed data

^a^Basic model adjusted for gestational age at assessment

^b^Maternal pregnancy-related factors model: basic model additionally adjusted for maternal ethnicity, age, parity, educational level, daily total energy intake, smoking, alcohol consumption and folic acid supplement use

^c^BMI model: maternal pregnancy-related factors model, additionally adjusted for maternal pre-pregnancy BMI

*p <0.05

### Sensitivity analyses

The sensitivity analyses using maternal early-pregnancy non-fasting insulin levels, instead of maternal early-pregnancy non-fasting glucose levels, showed that maternal early-pregnancy non-fasting insulin levels were largely similarly associated with fetal growth rates and fetal biometry measurements in each pregnancy period (ESM Table 5, ESM Figs 1 and 2). Based on comparison of the effect estimates per SDS increase in maternal early-pregnancy non-fasting glucose and insulin levels, the strength of the associations with fetal biometry measurements was also largely the same. Similar results to the main findings were found when we excluded women with pre-gestational diabetes mellitus or GDM and when we restricted our analyses to women of Dutch ethnicity only, women included in early pregnancy only and among term births only (ESM Table 6).

## Discussion

We observed that maternal early-pregnancy blood glucose levels across the full spectrum are associated with altered fetal growth patterns, characterised by decreased fetal growth rates in mid-pregnancy and increased fetal growth rates from late pregnancy onwards, and an increased risk of delivering a large-for-gestational-age infant. These associations were only partly explained by maternal pre-pregnancy BMI, and not by other maternal pregnancy-related factors.

### Interpretation of main findings

Maternal GDM and hyperglycaemia diagnosed in the second half of pregnancy are common and major risk factors for adverse birth outcomes. It is likely that women who develop GDM or hyperglycaemia later in pregnancy already have a suboptimal glucose metabolism preconceptionally or in early pregnancy, a critical period for embryonic and placental development [10, 11]. Despite well-known associations of maternal GDM with adverse birth outcomes, direct effects of a disturbed maternal glucose metabolism from early pregnancy onwards on fetal growth remain unclear. Fetuses of women with pre-gestational type 1 and type 2 diabetes mellitus are at increased risk for macrosomia at birth, but also for delayed growth during early pregnancy. This latter association may be due to poor glucose control already preconceptionally or very early in pregnancy [24–27]. The role of maternal glucose metabolism in early pregnancy in relation to fetal development and birth outcomes is not clear in women without overt diabetes.

Among women with GDM, it has been suggested that fetal growth is already abnormal preceding the diagnosis of GDM. Results from a cohort study among 533 women showed that, compared with those without GDM, those with GDM had smaller fetuses until 24 weeks of gestation, followed by accelerated fetal growth in late pregnancy [11]. A study among 4069 pregnant women showed that fetuses of women with GDM had increased abdominal circumference growth rates between 20 and 28 weeks of gestation, with the strongest effects among obese women [10]. Similarly, a prospective study among 741 black African women showed increased fetal abdominal circumference growth rates from mid-pregnancy onwards in women with GDM compared with those without GDM [28]. As maternal glucose levels before the diagnosis of GDM were not known in these studies, these findings are difficult to interpret in the context of the present study results. We observed that among non-diabetic women, higher maternal early-pregnancy non-fasting glucose levels across the full range were associated with decreased fetal growth rates in mid-pregnancy and increased fetal growth rates from late pregnancy onwards resulting in larger size at birth. These associations were independent of maternal ethnicity, a well-known risk factor for GDM and an important determinant for fetal growth. Also, other maternal pregnancy-related factors, including pre-pregnancy BMI, did not explain the observed associations. When we assessed each pregnancy period separately, we also observed that higher maternal early-pregnancy non-fasting glucose levels tended to be associated with smaller fetal biometry measurements in early pregnancy and mid-pregnancy, although for some measurements there was no significant association. Thus, our results suggest that already among non-diabetic women, higher maternal early-pregnancy non-fasting glucose levels within the normal range are related to altered fetal growth patterns, characterised by decreased fetal growth rates in mid-pregnancy and increased fetal growth rates from late pregnancy onwards. The presence of associations for all fetal biometry measurements suggests that maternal early-pregnancy non-fasting glucose levels affect both fetal fat development and skeletal growth.

Impaired maternal gestational glucose metabolism is a major risk factor for delivering a large-for-gestational-age infant, preterm birth and Caesarean delivery, with even stronger effects among overweight and obese women [2, 7, 29–32]. A retrospective study among more than 6000 women showed that higher maternal glucose levels at 9.5 weeks’ gestation were associated with an increased risk of delivering a large-for-gestational-age infant [3]. A case–control study among 2050 women with term deliveries observed an association of maternal early-pregnancy glucose levels with delivering large-for-gestational-age infants, independent of maternal BMI [31]. A large cohort among 46,000 women showed that higher maternal glucose levels between 10 and 24 weeks of gestation were associated with an increased risk of spontaneous preterm birth [33]. We observed that already a small increase in maternal early-pregnancy non-fasting glucose levels within the normal range was related to an increased risk of delivering a large-for-gestational-age infant, but a decreased risk of delivering a small-for-gestational-age infant. These associations were independent of maternal pre-pregnancy BMI. BMI is a measure of general adiposity, but does not provide any information on more specific fat compartments, such as visceral fat mass. Alterations in maternal visceral fat mass, which is more metabolically active*,* might explain part of the observed associations. In addition, our study population is a relatively lean population. The effect of maternal pre-pregnancy BMI on the observed associations might be stronger among more obese populations. We did not observe associations for preterm birth, Caesarean delivery or vacuum extraction. It seems likely that associations of maternal early-pregnancy non-fasting glucose levels with delivery complications are partly driven by size at birth as well as other maternal characteristics, such as maternal obesity. Even though we did observe that higher maternal early-pregnancy non-fasting glucose levels were associated with an increased risk of a large-for-gestational-age infant, the overall effect on birthweight in the full cohort was relatively small. This may partly explain the lack of associations with the delivery complications. These associations may be more apparent among higher-risk populations. Thus, our findings suggest that in a non-diabetic population, non-fasting glucose levels in early pregnancy already partly determine the risk of delivering a large-for-gestational-age infant.

The mechanisms underlying associations of maternal glucose metabolism with reduced fetal growth in the first half of pregnancy and increased fetal growth thereafter are not known. It has been suggested that impaired glucose control during early pregnancy negatively affects placental development, starting with impaired early placentation, which induces placental insufficiency and thereby early fetal growth restriction [11]. In response to the placental insufficiency, it has been suggested that the fetus may induce maternal hyperglycaemia to improve nutrient supply and growth during the second half of pregnancy via placental signalling [11]. It has also been hypothesised that hyperglycaemia in early pregnancy injures the development of the yolk sac, which is of great importance during the embryonic period, especially in nutrient transport towards the embryo. This may lead to impaired embryonic growth and development. When the yolk sac function is replaced by the placenta at the end of early pregnancy, hyperglycaemia together with increased transfer of other nutrients could induce an intrauterine environment that stimulates increased fetal adiposity and growth [2, 34].

Even though the observed effects for the associations of maternal blood glucose levels with altered fetal growth patterns and the risk of delivering a large-for-gestational-age infant are relatively small, they are important from an aetiological and preventive perspective. Importantly, we observed the adverse effects of maternal blood glucose levels across the full range of maternal early-pregnancy non-fasting glucose levels and not only at the diagnostic thresholds of impaired glucose metabolism. In addition, the observed associations were not explained by maternal sociodemographic factors or lifestyle factors, which suggests that potential intrauterine mechanisms may be involved. Current clinical practice is mainly focused on screening for GDM based on diagnostic thresholds of maternal glucose levels from mid-pregnancy onwards in higher-risk women. However, based on our findings, altered fetal development can already occur among non-diabetic women before mid-pregnancy, which is when screening for GDM and necessary interventions are currently implemented. Recent RCTs, which are considered the gold standard for studying causality, indicate that treatment of GDM and maternal hyperglycaemia with lifestyle adaptations from mid-pregnancy onwards leads to a decreased risk of adverse birth outcomes compared with no treatment [35–37]. Based on our findings, future RCTs should focus on glucose screening and treatment from preconception and early pregnancy onwards to further improve pregnancy outcomes, among higher-risk populations such as overweight and obese women and possibly also among lower-risk populations. These studies should assess the effects of lifestyle interventions that keep an adequate balance between reducing maternal blood glucose levels without inducing hypoglycaemia and preventing hyperglycaemia. These interventional studies from preconception and early pregnancy onwards will not only provide important novel insights into the effectiveness of these interventions, but also into the causality of the observed associations of maternal early-pregnancy non-fasting glucose levels with altered fetal growth and adverse birth outcomes.

### Methodological considerations

Major strengths of this study are the population-based prospective design with a large sample size with information on maternal blood glucose levels and fetal growth throughout pregnancy. The response rate at baseline was 61%. The non-response at baseline would lead to biased effect estimates if associations were different between those included and not included in the analyses, but this seems unlikely. We had a relatively small number of cases of GDM, which indicates a selection towards a non-diabetic population and might affect the generalisability of our findings. The observed associations might be stronger among higher-risk populations. Information on GDM was obtained from medical records after delivery. Accurate diagnosis of GDM is difficult. A fasting glucose greater that 7.0 mmol/l might also represent preexisting diabetes mellitus and a fasting glucose between 6.1 and 6.9 mmol/l might also represent impaired glucose tolerance, instead of GDM. Unfortunately, in our study, glucose testing for diagnosis of GDM was not done for all women for study purposes and no data were available on glucose tolerance before pregnancy. Further studies are needed to replicate our findings among more high-risk populations, including women with impaired glucose tolerance from preconception and early pregnancy onwards and women at risk to develop GDM. We only measured maternal glucose levels once in early pregnancy. However, it has been suggested that impaired glucose control in early pregnancy persists throughout pregnancy [38]. The fasting time before venous puncture was limited to 30 min, due to which we consider our samples non-fasting samples. We were not able to collect blood samples after a longer fasting period due to the design of the study. The blood samples were collected in a non-fasting state at different time-points during the day, depending on time of the study visit. Since glucose levels shift very easily during the day and are sensitive towards carbohydrate intake, this may have led to non-differential misclassification of what would be classified as high- or low-glucose levels and an underestimation of the observed effect estimates. We also did not have information on 1 h and 2 h postprandial glucose levels available. However, it has been suggested that maternal fasting glucose levels, postprandial glucose levels and non-fasting random samples are appropriate measures of maternal glucose metabolism and are related to adverse birth outcomes [2, 7]. Non-fasting blood values may better reflect the normal physiological state in pregnant women [4, 31]. Further studies are needed to replicate our findings using more detailed maternal glucose measurements, including fasting glucose levels and detailed postprandial glucose measurements. Although we included many covariates, there still might be some residual confounding, as in any observational study.

## Conclusions

Maternal early-pregnancy non-fasting blood glucose level is associated with altered fetal growth patterns, characterised by decreased fetal growth rates in mid-pregnancy and increased fetal growth rates from late pregnancy onwards, and an increased risk of delivering a large-for-gestational-age infant. These associations are only partly explained by maternal pre-pregnancy BMI. Instead of targeting maternal glucose metabolism in the second half of pregnancy as in current clinical practice, future preventive strategies need to focus on screening for an impaired maternal glucose metabolism from preconception and early pregnancy onwards to improve fetal growth and birth outcomes.

## Electronic supplementary material

ESM (PDF 216 kb)

## Data Availability

Data are available by request from the corresponding author.

## Abbreviations

GDM: Gestational diabetes mellitus
SDS: SD score
