# Supplementary material for: High maternal early-pregnancy blood glucose levels are associated with altered fetal growth and increased risk of adverse birth outcomes
Source: Diabetologia. 2019 Aug 8;62(10):1880–90. doi: 10.1007/s00125-019-4957-3 (PMC6731335; doi:10.1007/s00125-019-4957-3)
Supplement: Supplementary file 1 — (PDF 216 kb) [file 125_2019_4957_MOESM1_ESM.pdf]

Electronic supplementary material

High maternal early-pregnancy blood glucose levels are associated with altered fetal growth and increased risk of adverse birth outcomes

Madelon L. Geurtsen, Eef E.L. van Soest, Ellis Voerman,

Eric A. P. Steegers, Vincent W.V. Jaddoe, Romy Gaillard

CONTENTS

ESM Table 1 Non-response analyses

ESM Table 2 Associations of maternal early-pregnancy glucose levels in mmol/l with fetal biometry measurements

ESM Table 3 Associations of maternal early-pregnancy glucose levels in mmol/l with absolute fetal biometry measurements

ESM Table 4 Associations of maternal early-pregnancy glucose levels in mmol/l with the risks of adverse birth outcomes

ESM Table 5 Associations of maternal early-pregnancy glucose and insulin levels in SD with fetal biometry measurements

ESM Table 6 Sensitivity analyses of fetal biometry measurements

ESM Fig. 1 Maternal early-pregnancy glucose levels in SD and longitudinal fetal growth rates

ESM Fig. 2 Maternal early-pregnancy insulin levels in SD and longitudinal fetal growth rates

ESM Table 1 Non-response analyses

	**Participants** $N = 6116$	**Non-participants** $n = 2763$	**P-value**[a]
Maternal characteristics			
Age, years	29.8 ± 5.1	29.4 ± 5.8	0.004
Height, cm	167.5 ± 7.4	166.2 ± 7.4	<0.001
Pre-pregnancy weight, kg	64.0 (48.0–99.7)	63.0 (48.0–100.0)	0.386
Pre-pregnancy BMI, kg/m^2	22.6 (18.0–34.7)	22.8 (17.8–36.0)	0.022
Gestational age at intake, weeks	13.2 (9.6–17.6)	19.6 (10.8–31.4)	<0.001
Parity (nulliparous)	3474 (57.3)	1387 (51.6)	<0.001
Ethnicity			
Dutch	3083 (52.2)	1013 (40.4)	<0.001
European	496 (8.4)	175 (7.0)	
Cape Verdean	245 (4.2)	110 (4.4)	
Moroccan	353 (6.0)	227 (9.1)	
Dutch Antillean	171 (2.9)	124 (5.0)	
Surinamese	506 (8.6)	258 (10.3)	
Turkish	474 (8.1)	298 (11.9)	
Other	545 (9.3)	300 (12.0)	
Education, higher	2550 (44.9)	828 (34.8)	<0.001
Total calorie intake, kJ	486 ± 134	489 (141)	0.488
Folic acid use			
No	1183 (25.3)	745 (39.4)	<0.001
Start first 10 weeks	1491 (31.9)	549 (29.1)	
Start periconceptional	1997 (42.8)	595 (31.5)	
Smoking during pregnancy, continued	1012 (18.6)	428 (18.6)	0.963
Alcohol use during pregnancy, continued	2095 (39.0)	691 (30.5)	<0.001
Gestational hypertensive disorders			
Pre-eclampsia	127 (2.2)	60 (2.4)	0.677
Gestational hypertension	234 (4.1)	84 (3.3)	0.107
Pre-gestational diabetes mellitus	24 (0.5)	9 (0.4)	0.754
GDM	62 (1.1)	29 (1.1)	0.752
Birth Characteristics			
Males	3100 (50.7)	1301(49.9)	0.483
Gestational age, weeks	40.1 (35.6–42.3)	40.0 (34.7–42.4)	<0.001
Preterm birth[b]	310 ± 5.1	199 ± 7.6	<0.001
Small for gestational age[c]	606 (10.0)	254 (9.9)	0.894
Large for gestational age[d]	606 (10.0)	234 (8.5)	0.211
Caesarean delivery	692 (14.5)	277 (13.8)	0.452
Vacuum extraction	774 (15.9)	277 (13.8)	0.025

Values are numbers (%), means ± SD or medians (95% range)

[a] Differences in subject characteristics between participants and non-participants were evaluated using one-way ANOVA test for continuous variables and chi-square tests for categorical variables

[b] Preterm birth is defined as <37 weeks' gestation

[c] Small for gestational age is defined as <10th percentile of age-and sex-adjusted birth weight

[d] Large for gestational age is defined as >90th percentile of age-and sex-adjusted birth weight

ESM Table 2 Associations of maternal early-pregnancy glucose levels in mmol/l with fetal biometry measurements

**Maternal early-pregnancy glucose levels in mmol/l**				
Early pregnancy	Crown-rump Length			
Basic model[a]	-0.02 (-0.08, 0.03)			
Ethnicity model[b]	-0.02 (-0.08, 0.03)			
Maternal pregnancy-related factors model[c]	-0.04 (-0.09, 0.02)			
BMI model[d]	-0.04 (-0.09, 0.02)			
Mid-pregnancy	Head circumference	Abdominal circumference	Femur length	Estimated fetal weight
Basic model[a]	-0.03 (-0.06, 0.00)*	-0.05 (-0.08, -0.02)*	0.01 (-0.02, 0.04)	-0.02 (-0.05, 0.01)
Ethnicity model[b]	-0.03 (-0.06, 0.00)	-0.04 (-0.07, -0.01)*	0.01 (-0.02, 0.04)	-0.02 (-0.05, 0.01)
Maternal pregnancy-related factors model[c]	-0.03 (-0.06, 0.00)	-0.05 (-0.08, -0.02)*	0.01 (-0.02, 0.04)	-0.02 (-0.05, 0.01)
BMI model[d]	-0.04 (-0.07, -0.01)*	-0.06 (-0.09, -0.02)*	-0.01 (-0.04, 0.02)	-0.04 (-0.07, -0.01)*
Late pregnancy	Head circumference	Abdominal circumference	Femur length	Estimated fetal weight
Basic model[a]	0.03 (-0.01, 0.06)	0.05 (0.02, 0.08)*	0.04 (0.01, 0.07)*	0.05 (0.02, 0.08)*
Ethnicity model[b]	0.03 (0.00, 0.06)*	0.05 (0.02, 0.08)*	0.04 (0.01, 0.07)*	0.05 (0.02, 0.08)*
Maternal pregnancy-related factors model[c]	0.03 (0.00, 0.06)	0.04 (0.01, 0.07)*	0.03 (0.00, 0.06)*	0.04 (0.01, 0.07)*
BMI model[d]	0.02 (-0.02, 0.05)	0.02 (-0.01, 0.05)	0.01 (-0.02, 0.04)	0.02 (-0.01, 0.05)
Birth	Head circumference	Abdominal circumference	Length	Weight
Basic model[a]	0.04 (0.00, 0,09)	NA	0.04 (-0.01, 0.08)	0.07 (0.04, 0.11)*
Ethnicity model[b]	0.05 (0.01, 0,10)*	NA	0.04 (0.00, 0.09)*	0.08 (0.05, 0.11)*
Maternal pregnancy-related factors model[c]	0.04 (0.00, 0,09)	NA	0.04 (-0.01, 0.08)	0.07 (0.04, 0.10)*
BMI model[d]	0.04 (-0.01, 0.08)	NA	0.02 (-0.02, 0.07)	0.05 (0.02, 0.08)*

Data are SDS values (95% CI) from linear regression models that reflect the differences in growth characteristics per 1 mmol/l in maternal early-pregnancy glucose levels. Analyses with crown-rump length were based on subgroup analyses (n = 1470). Estimates are from multiple imputed data

[a] Basic model adjusted for gestational age at assessment

[b] Ethnicity model: basic model additionally adjusted for maternal ethnicity

[c] Maternal pregnancy-related factors model: ethnicity model additionally adjusted for maternal age, parity, educational level, daily total calorie intake, smoking, alcohol consumption and folic acid supplement use

[d] BMI model: maternal pregnancy-related factors model additionally adjusted for maternal prepregnancy BMI

*p<0.05

Abbreviations: NA, not available

ESM Table 3 Associations of maternal early-pregnancy glucose levels in mmol/l with absolute fetal biometry measurements

**Maternal early-pregnancy glucose levels in mmol/l**	**Head circumference (mm) / at birth (cm)**	**Abdominal circumference (mm) / NA**	**Femur length (mm) / length at birth (cm)**	**Estimated fetal weight (gram) / weight at birth (gram)**
Mid-pregnancy	-0.32 (-0.72, 0.08)	-0.51 (-0.92, -0.10)*	-0.02 (-0.11, 0.08)	-1.95 (-4.48, 0.57)
Late pregnancy	0.26 (-0.12, 0.63)	0.55 (0.06, 1.05)*	0.08 (-0.01, 0.17)	8.46 (0.86, 16.06)*
Birth	0.04 (-0.03, 0.11)	NA	0.07 (-0.02, 0.15)	25.38 (8.98, 41.79)*

Data are exact values (95% CI) from linear regression models that reflect the differences in growth characteristics per 1 mmol/l in maternal early-pregnancy glucose levels. Estimates are from multiple imputed data. Models are adjusted according maternal pregnancy-related factors model adjusted for gestational age at assessment, maternal ethnicity, age, parity, educatoinal level, daily total calorie intake, smoking, alcohol consumption and folic acid supplement use

*p<0.05

Abbreviations: NA, not available.

ESM Table 4 Associations of maternal early-pregnancy glucose levels in mmol/l with the risks of adverse birth outcomes

**Maternal early-pregnancy glucose levels0 mmol/l**	**Small size for gestational age at birth**	**Large size for gestational age at birth**	**Preterm birth**	**Caesarean delivery**	**Vacuum extraction**
Basic model[a]	0.89 (0.80, 0.98)*	1.27 (1.16, 1.40)*	1.08 (0.95, 1.24)	1.12 (0.98, 1.27)	0.99 (0.90, 1.09)
Ethnicity model[b]	0.87 (0.79, 0.97)*	1.29 (1.18, 1.42)*	1.08 (0.94, 1.23)	1.12 (0.99, 1.27)	1.00 (0.91, 1.11)
Maternal pregnancy-related factors model[c]	0.88 (0.79, 0.98)*	1.28 (1.16, 1.41)*	1.08 (0.94, 1.23)	1.11 (1.00, 1.23)	1.01 (0.91, 1.12)
BMI model[d]	0.91 (0.82, 1.02)	1.21 (1.10, 1.34)*	1.06 (0.92, 1.21)	1.09 (0.99, 1.20)	1.01 (0.90, 1.12)

Values are ORs (95% CI) from logistic regression models that reflect the differences in risks of adverse birth outcomes per 1 mmol/l in maternal early-pregnancy glucose levels. Estimates are from multiple imputed data

[a] Basic model adjusted for gestational age at assesment

[b] Ethnicity model: basic model additionally adjusted for maternal ethnicity

[c] Maternal pregnancy-related factors model: ethnicity model additionally adjusted for maternal age, parity, educational level, daily total calorie intake, smoking, alcohol consumption and folic acid supplement use

[d] BMI model: maternal pregnancy-related factors model additionally adjusted for maternal prepregnancy BMI

*p<0.05

ESM Table 5 Associations of maternal early-pregnancy glucose and insulin levels in SD with fetal biometry measurements

**Early pregnancy**	**Crown-rump Length**			
Glucose (SD)	-0.02 (-0.07, 0.03)			
Insulin (SD)	-0.02 (-0.07, 0.04)			
**Mid-pregnancy**	**Head circumference**	**Abdominal circumference**	**Femur length**	**Estimated fetal weight**
Glucose (SD)	-0.02 (-0.05, 0.00)	-0.04 (-0.07, -0.02)*	0.01 (-0.02, 0.03)	-0.02 (-0.05, 0.00)
Insulin (SD)	-0.01 (-0.04, 0.02)	-0.02 (-0.04, 0.01)	0.04 (0.01, 0.06)*	0.01 (-0.02, 0.03)
**Late pregnancy**	**Head circumference**	**Abdominal circumference**	**Femur length**	**Estimated fetal weight**
Glucose (SD)	0.02 (0.00, 0.05)	0.03 (0.01, 0.06)*	0.03 (0.00, 0.05)*	0.04 (0.01, 0.06)*
Insulin (SD)	0.00 (-0.03, 0.03)	0.04 (0.01, 0.06)*	0.06 (0.04, 0.09)*	0.05 (0.03, 0.08)*
**Birth**	**Head circumference**	**Abdominal circumference**	**Length**	**Weight**
Glucose (SD)	0.04 (0.00, 0.08)	NA	0.03 (0.00, 0.07)	0.06 (0.04, 0.09)*
Insulin (SD)	0.06 (0.01, 0.09)*	NA	0.08 (0.04, 0.12)*	0.06 (0.03, 0.08)*

Data are SDS values (95% CI) from linear regression models that reflect the differences in growth characteristics per 1 SD change in maternal early-pregnancy glucose and insulin levels. Insulin was logtransformed before the construction of SD score. Analyses with crown-rump length were based on subgroup analyses (n = 1470). Estimates are from multiple imputed data. Models are adjusted according maternal pregnancy-related factors model adjusted for gestational age at assessment, maternal ethnicity, age, parity, educational level, daily total calorie intake, smoking, alcohol consumption and folic acid supplement use

*p <0.05

Abbreviations: NA, not available

ESM Table 6 Sensitivity analyses of fetal biometry measurements

**Early pregnancy**	**Crown-rump Length**			
Main model	-0.04 (-0.09, 0.02)			
No pre-gestational diabetes mellitus	-0.05 (-0.10, 0.01)			
No gestational diabetes mellitus	-0.05 (-0.11, 0.01)			
Dutch ethnicity	-0.04 (-0.11, 0.03)			
Included <14 weeks gestation	-0.04 (-0.10, 0.02)			
No preterm	-0.05 (-0.11, 0.01)			
**Mid-pregnancy**	**Head circumference**	**Abdominal circumference**	**Femur length**	**Estimated fetal weight**
Main model	-0.03 (-0.06, 0.00)	-0.05 (-0.08, -0.02)*	0.01 (-0.02, 0.04)	-0.02 (-0.05, 0.01)
No pre-gestational diabetes mellitus	-0.02 (-0.06, 0.01)	-0.05 (-0.08, -0.02)*	0.01 (-0.03, 0.04)	-0.03 (-0.06, 0.00)
No gestational diabetes mellitus	-0.03 (-0.06, 0.00)	-0.05 (-0.08, -0.02)*	0.01 (-0.02, 0.04)	-0.03 (-0.06, 0.00)
Dutch ethnicity	-0.04 (-0.09, 0.00)	-0.07 (-0.12, -0.03)*	0.01 (-0.03, 0.05)	-0.04 (-0.08, 0.00)
Included <14 weeks gestation	0.00 (-0.02, 0.02)	-0.04 (-0.07, -0.01)*	0.03 (-0.01, 0.06)	-0.01 (-0.04, 0.03)
No preterm	-0.03 (-0.06, 0.01)	-0.05 (-0.08, -0.02)*	0.00 (-0.03, 0.03)	-0.03 (-0.06, 0.00)
**Late pregnancy**	**Head circumference**	**Abdominal circumference**	**Femur length**	**Estimated fetal weight**
Main model	0.03 (0.00, 0.06)	0.04 (0.01, 0.07)*	0.03 (0.00, 0.06)*	0.04 (0.01, 0.07)*
No pre-gestational diabetes mellitus	0.03 (0.00, 0.06)	0.03 (0.00, 0.06)	0.02 (-0.01, 0.06)	0.03 (0.00, 0.07)*
No gestational diabetes mellitus	0.02 (-0.01, 0.05)	0.03 (0.00, 0.06)*	0.03 (0.00, 0.06)*	0.04 (0.01, 0.07)*
Dutch ethnicity	0.00 (-0.05, 0.04)	0.02 (-0.02, 0.07)	0.03 (-0.01, 0.07)	0.03 (-0.01, 0.07)
Included <14 weeks gestation	0.04 (0.00, 0.08)*	0.04 (0.00, 0.08)*	0.06 (0.02, 0.10)*	0.06 (0.02, 0.09)*
No preterm	0.03 (-0.01, 0.06)	0.03 (0.00, 0.07)*	0.03 (0.00, 0.06)*	0.04 (0.01, 0.07)*
**Birth**	**Head circumference**	**Abdominal circumference**	**Length**	**Weight**
Main model	0.04 (0.00, 0,09)	NA	0.04 (-0.01, 0.08)	0.07 (0.04, 0.10)*
No pre-gestational diabetes mellitus	0.03 (-0.02, 0,08)	NA	0.04 (0.00, 0.09)	0.07 (0.04, 0.10)*
No gestational diabetes mellitus	0.04 (-0.01, 0.09)	NA	0.04 (-0.01, 0.08)	0.06 (0.04, 0.10)*
Dutch ethnicity	0.02 (-0.04, 0.08)	NA	0.02 (-0.03, 0.08)	0.06 (0.01, 0.10)*
Included <14 weeks gestation	0.02 (-0.04, 0.08)	NA	0.03 (-0.02, 0.08)	0.09 (0.05, 0.12)*
No preterm	0.05 (0.00, 0,10)*	NA	0.04 (-0.01, 0.08)	0.07 (0.04, 0.10)*

Data are SDS values (95% CI) from linear regression models that reflect the differences in growth characteristics per 1 mmol/l in maternal early-pregnancy glucose levels. Analyses with crown-rump length were based on subgroup analyses (*n* = 1470). Estimates are from multiple imputed data. Models are adjusted according maternal pregnancy-related factors model adjusted for gestational age at assessment, maternal ethnicity, age, parity, educational level, daily total calorie intake, smoking, alcohol consumption and folic acid supplement use

*$p < 0.05$

Abbreviations: NA, not available

ESM Fig. 1

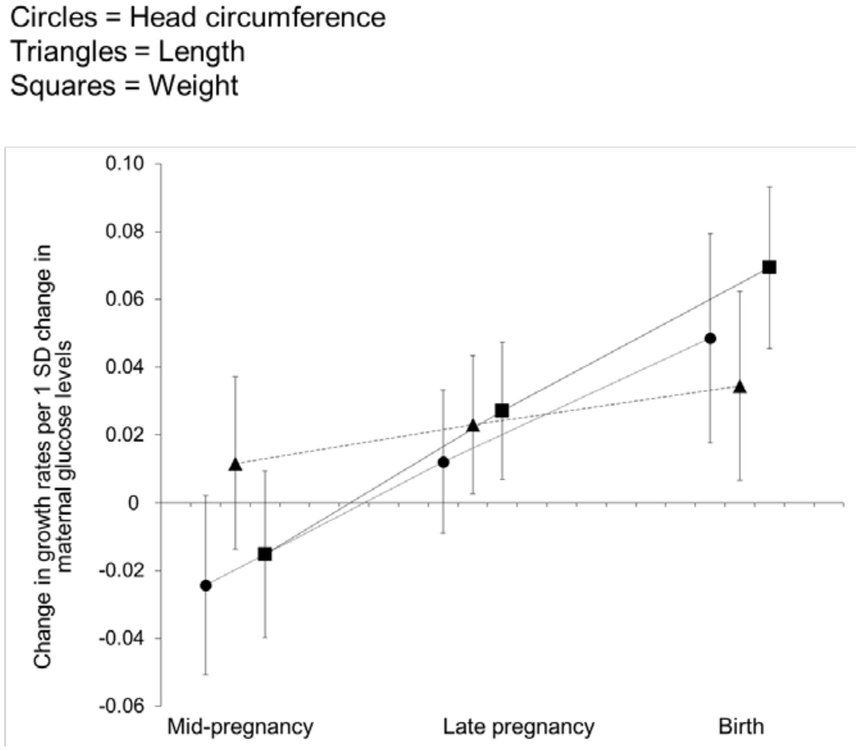

Maternal early-pregnancy glucose levels in SD and longitudinal fetal growth rates. Data are SDS values (95% CI) from repeated measurement regression models that reflect the differences in gestational age-adjusted growth rates in SDS of head circumference (circles), length (triangles), and weight (squares) at mid-pregnancy, late pregnancy and at birth per 1 SD change in maternal early-pregnancy glucose levels. As a measure of skeletal length growth from mid-pregnancy onwards, we used fetal femur length SDS in mid-pregnancy and late pregnancy and total body length SDS at birth within the repeated measurements model. All fetal biometry measurements for each pregnancy period were taken at the same time point. The models were adjusted for gestational age at intake. *p* value for interaction with gestational age for all models <0.05.

ESM Fig. 2

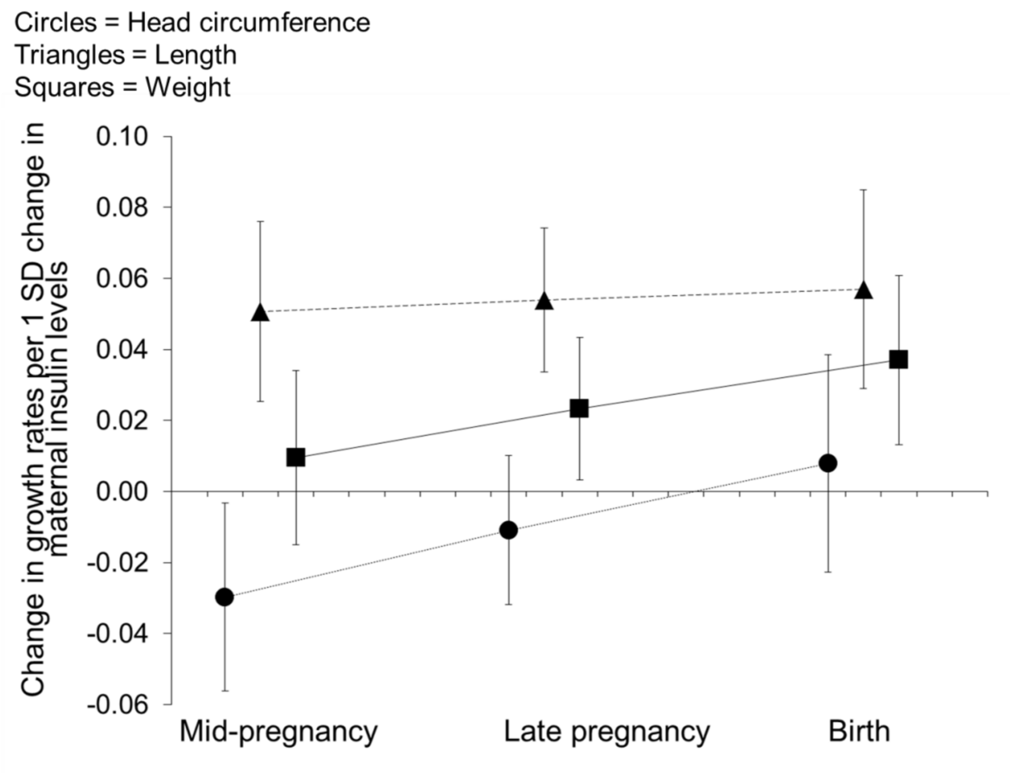

Maternal early-pregnancy insulin levels in SD and longitudinal fetal growth rates. Data are SDS values (95% CI) from repeated measurement regression models that reflect the differences in gestational age-adjusted growth rates in SDS of head circumference (circles), length (triangles), and weight (squares) at mid-pregnancy, late pregnancy and at birth per 1 SD change in maternal early-pregnancy insulin levels. As a measure of skeletal length growth from mid-pregnancy onwards, we used fetal femur length SDS in mid-pregnancy and late pregnancy and total body length SDS at birth within the repeated measurements model. All fetal biometry measurements for each pregnancy period were taken at the same time point. The models were adjusted for gestational age at intake. *p* value for interaction with gestational age for all models <0.05.
